# The Current State and Future of CRISPR-Cas9 gRNA Design Tools

**DOI:** 10.3389/fphar.2018.00749

**Published:** 2018-07-12

**Authors:** Laurence O. W. Wilson, Aidan R. O’Brien, Denis C. Bauer

**Affiliations:** ^1^Commonwealth Scientific and Industrial Research Organisation, Sydney, NSW, Australia; ^2^Department of Immunology and Infectious Disease, John Curtin School of Medical Research, Acton, ACT, Australia

**Keywords:** CRISPR-Cas9, bioinformatics, off-target finder, activity prediction, chromatin, machine learning

## Abstract

Recent years have seen the development of computational tools to assist researchers in performing CRISPR-Cas9 experiment optimally. More specifically, these tools aim to maximize on-target activity (guide efficiency) while also minimizing potential off-target effects (guide specificity) by analyzing the features of the target site. Nonetheless, currently available tools cannot robustly predict experimental success as prediction accuracy depends on the approximations of the underlying model and how closely the experimental setup matches the data the model was trained on. Here, we present an overview of the available computational tools, their current limitations and future considerations. We discuss new trends around personalized health by taking genomic variants into account when predicting target sites as well as discussing other governing factors that can improve prediction accuracy.

## Introduction

The CRISPR-Cas9 system allows for targeted editing of DNA *in vitro*. The system is targeted to the DNA via association with a guide RNA (gRNA) molecule, which binds to the targeted DNA through base complementarity and enables precise DNA cleavage (Jinek et al., 2013). This cleavage is then repaired via various pathways, which can be exploited for different outcomes (Kim and Kim, 2014). Knockouts can be achieved through error prone repair via the Non-homologous End Joining pathway, which can introduce mutations and disrupt gene function. Targeted integration of a sequence (called a knock-in) can be achieved via the Homology Directed Repair pathway, which uses a provided DNA template to repair the cleavage. Activation or repression of a gene can be achieved by targeting catalytically inert Cas9 fused to a transcription activator or repressor to the promoter (La Russa and Qi, 2015). All of these approaches require the accurate and efficient targeting of the CRISPR-Cas9 system to the desired location. The success of an experiment using the CRISPR-Cas9 system therefore hinges on the correct identification of the optimal target-site and subsequent design of the complimentary gRNA (Mali et al., 2013; Chari et al., 2015). While databases of validated gRNAs exist for various genomes [e.g., Cas-Database (Park et al., 2016) for knockout applications and (Horlbeck et al., 2016a) for gene activation/repression], these libraries are generic and may not be well-suited for specific research purposes. The design of custom gRNAs is hence frequently required.

A successful gRNA must maximize on-target activity (guide efficiency) while also minimizing potential off-target effects (guide specificity). Balancing these two requirements can be a combinatorial challenging task and as a result, significant effort in the recent years has been focused on developing computational tools to assist in the design of gRNAs. These tools are designed to assist researchers in the selection of best target sites by helping them exclude undesirable targets based on predicted low efficiency or a high potential for off-target effects. Here, we present an overview of the development of tools for the design of CRISPR-Cas9 gRNAs, their current limitations and future considerations.

## Predicting On-Target Activity

Initially, CRISPR-Cas9 was thought to be able to target any 20 base-pair sequence that was flanked by a protospacer adjacent motif (PAM). Different Cas and related enzymes target different PAMs, and there is ongoing researching into designing enzymes with specific PAM recognition ability (Cebrian-Serrano and Davies, 2017). However, the most commonly used SpCas9, and the focus of this review, targets an NGG motif. As such, early tools for target site selection were simple pattern recognition programs that identified instances of this motif (Upadhyay and Sharma, 2014; Xie et al., 2014; Zhu et al., 2014). In some cases, information about where in a gene the target site fell (e.g., within an intron or exon) was also incorporated, allowing researchers to draw some conclusions on the likelihood of a functional effect. However, subsequent studies showed that CRISPR-Cas9 displayed a wide variety of activities across different target sites, leading to the conclusion that some target sites are inherently more effective (Jinek et al., 2012, 2013; Cong et al., 2013; Fu et al., 2013, 2014; Mali et al., 2013; Yang et al., 2013; Doench et al., 2014; Koike-Yusa et al., 2014; Shalem et al., 2014; Wang et al., 2014; Chari et al., 2015; Moreno-Mateos et al., 2015).

This discovery led to a series of large-scale screens of CRISPR-Cas9 activity across a variety of target sites and organisms, aimed at identifying what features contributed to targeting efficiency (Hsu et al., 2013; Doench et al., 2014, 2016; Chari et al., 2015; Moreno-Mateos et al., 2015; Horlbeck et al., 2016b). These studies helped identify some key rules for optimizing gRNA design. This include avoiding poly-T sequences, limiting the GC content and a G immediately upstream of the PAM (i.e., an GNGG motif) (Ren et al., 2014; Shalem et al., 2014; Wong et al., 2015). Building on this research, computational methods were created for predicting on-target activity. The initial studies focused on the contribution of the target site sequence, by measuring the activity of 1000s of target sites. These studies differed in how they defined the target sites, with some considering only the 20 bp target sequence (Chari et al., 2015) while others included the PAM and flanking sequence (Doench et al., 2014; Moreno-Mateos et al., 2015; Wong et al., 2015). They also differed in how they represented the target site to the mathematical model, i.e., the feature space. The studies used different combinations of position specific nucleotides and dinucleotides, global nucleotide counts, GC content, etc. More recent studies have also begun to include non-sequence information, such as thermodynamic stability of the gRNA and position of the cut site relative to the transcription start site (TSS) (Doench et al., 2014; Wong et al., 2015; Horlbeck et al., 2016b).

The differences in experimental design means that each study produced a unique predictive model, with different rules for CRISPR-Cas9 activity. Supplementary Table 1 presents a selection of tools that demonstrate the variety of data types, features, and model implementations used. Despite the differences in the model, however, certain key features were repeatedly found to be important. These include position-specific nucleotides, such as a G preceding the PAM being a strong indicator of CRISPR-Cas9 activity, or global variables such as GC content and gRNA melting temperature were consistently reported as being important (Wong et al., 2015). Comparing the distribution of important features along the target site, the majority are found within the ∼10–12 bp adjacent to the PAM, a region that has become known as the seed region (Liu et al., 2016). This region is typically thought to be critical for CRISPR-Cas9 activity, as this region binds the DNA first following recognition of the PAM (Farasat and Salis, 2016; Shibata et al., 2017).

The models also differed in what machine learning technique was used in their construction. While predicting activity using linear regression showed some success (Moreno-Mateos et al., 2015), the more successful models used more complex approaches such as Random Forest (Wilson et al., 2018) and Support Vector Machines (Chari et al., 2015; Wong et al., 2015; Doench et al., 2016), which consider interactions between the individual features (McKinney et al., 2006). The success of these more complex models suggests that there is no single feature that governs activity, but rather a combination of interactions.

Despite the extensive training of the models, the accuracy of their predictions varies widely. A recent review of different on-target efficiency models found that no model was consistently accurate across a number of independent dataset, recording high accuracy only when tested on the original training dataset (Haeussler et al., 2016). This discrepancy is likely due to the differences in how the various studies conducted their experiments. Consistent with this, a recent review found that predictive models performed best when the CRISPR-Cas9 expression system matched the one used in the training dataset (Haeussler et al., 2016). This would suggest that experimental conditions do affect the final model.

This same study also found that the method used to transcribe the gRNAs may also influence activity prediction. Typically, gRNAs are transcribed in cells from a U6 promoter or *in vitro* from a T7 promoter (Zhang et al., 2017). These promoters have differing transcription requirements, such as different polymerases and a G (for the U6 promoter) or GG (for the T7 promoter) at the TSS. These differences appear to influence any predictive model, with models performing better when applied to datasets that use the same promoter as what was used in the model’s training set (Haeussler et al., 2016). Currently, no one predictive model is able to account for gRNA transcription method. Some pipelines (such as CRISPOR), use multiple predictive models allowing researchers to select the most appropriate score (Haeussler et al., 2016).

It is also highly likely that the manner in which CRISPR-Cas9 activity is measured impacts the final model. There is currently no consensus in the literature in how CRISPR-Cas9 activity should be measured. Some studies measure activity by the rate at which mutations are introduced through sequencing the target site (Chari et al., 2015; Moreno-Mateos et al., 2015), while others measure activity by the size of the phenotypic change (such as drug resistance, cell viability, or protein expression) (Doench et al., 2014, 2016; Horlbeck et al., 2016b). While measuring activity via sequencing may prove a more direct measurement, it is also a costlier approach and does not provide information about whether the induced mutations are functional. Conversely, while phenotypic screens are easier to perform at scale they rely on the CRISPR-Cas9 introducing functional mutations, which may in turn lead to increase in false-negatives (i.e., mutations that do not cause a functional effect). These differences in experimental design likely translate to differences in the model.

Doench et al. (2016) reported that two of the most important variables for predicting CRISPR-Cas9 activity are the position of the target site relative to the TSS and position within the protein. However, this study was performed using a dataset that reported CRISPR-Cas9 activity based on a combination of changes in drug resistance and expression of cell-surface proteins. Given mutations near the TSS of a gene are more likely to induce a functional change, it is highly likely that the importance of target position is inflated in a phenotypic screen. In fact, a recent study comparing the impact of different training sets found that training a predictive model using sequencing-based measurements of CRISPR-Cas9 activity yields more generalizable predictions (Wilson et al., 2018). Phenotypic-trained models are governed by features such as position of the target site relative to TSS and do not generalize to other datasets.

Because the training dataset has such a strong influence on the final predictive model, it is therefore critical to know on what a model was trained before use. As a rule of thumb, phenotypic-trained models will be better suited to identifying target sites that induce functional changes but are limited to experiments with the same condition as the training set. In contrast, sequencing-based models are more universally applicable, but are only capable of predicting genotype changes not their functional result.

## Predicting Off-Target Activity

Identifying potential off-target sites is typically done by repurposing computational tools used for high-throughput sequencing read alignment. Here, the target site is treated as a read and realigned back to the reference genome in order to identify similar locations that may be inadvertently targeted by the CRISPR-Cas9. Alignment of the short target sequences is typically achieved using tools such as Bowtie and BWA, which are better suited for handling short sequences compared to other traditional tools such as BLAST.

These repurposed tools, however, are not the optimal solution for this problem. Searching for potential off-target sites requires the identification of small sequence motifs (20 bp + PAM) with often many mismatches. Traditional alignment tools are not equipped to identify such small, divergent sequences. Typically, Bowtie alignments allow only up to three mismatches while BWA allows up to 5, resulting in more divergent off-targets being missed. In fact comparison of off-target identification pipelines with experimentally validated CRISPR-Cas9 off-targets shows that these traditional alignment methods not only miss the high-mismatch off-target but even some with only one mismatch (Tsai et al., 2015; Doench et al., 2016), suggesting these tools are generally poorly suited for this problem.

Implementation of new alignment methods, such as bi-directional alignments (Canzar and Salzberg, 2017), will be required to accurately identify all potential off-targets. Typically, aligners work by first matching a small portion of the query sequence (known as the seed) and then extending the seed out in a direction and testing the match. Bi-directional aligners work by extending the initial seed region in both directions. Using these more powerful alignment tools will be important for correctly identifying all potential off-targets.

Further complicating the matter is that not every putative off-target is actually functional (i.e., off-targets that are actually cleaved by CRISPR-Cas9). As such, naive alignment methods hence return a large number of false-positives, potentially leading to the erroneous disqualification of the optimal target site.

A recent study comparing experimentally validated off-targets and those predicted by alignment tools, showed that the prediction tools over-estimate the number of potential off-targets by up to 10-fold (Cameron et al., 2017). In order to reduce the number of false-positive predictions, off-target predictors often limit potential off-targets to a maximum number of mismatches and only very specific PAMs (Bae et al., 2014b). However, experimental studies, have shown that off-targets can differ significantly from the original target site, meaning this approach often results in false-negatives (Tsai et al., 2015, 2017; Cameron et al., 2017). Predictive programs therefore need to balance the false-positives and false-negatives. To compensate for this, several studies have developed scoring algorithms, which attempt to predict the activity of a potential off-target so that false-positives can be filtered out.

The two most popular scoring methods are the MIT-Broad score (Hsu et al., 2013) and the CFD score (Doench et al., 2016). Both of these scoring algorithms are based on “synthetic” datasets, whereby a series of gRNAs targeting a specific dataset were mutated such that every one, two, and three base mismatch combination was represented. The ability of the gRNAs to cleave the target site were then measured, and the results used to construct a Linear Regression algorithm to score the off-target sites. Despite the theory behind both methods being similar, they differ in how the final model is constructed. While the MIT-Broad algorithm considers only the 20 bp target sequence (i.e., does not included the PAM), the CFD score takes the PAM sequence into account, scoring target sites as less active if they possess non-canonical PAMs. A recent comparison of the method tested their ability to accurately predict the off-target activity of different experimental datasets and concluded that the CFD score performed the best (Haeussler et al., 2016). These methods however, are limited in the features they consider, focusing predominantly on the number and position of mismatches. Two recently developed off-target methods Elevation (Listgarten et al., 2018) and CRISTA (Abadi et al., 2017) expand the feature set, including features such as gRNA secondary structure, genomic location and overlap with other features of interest such as DNase 1 Hypersensitive sites. These models are also capable of distinguishing between mismatches that occur through wobble pairing, and those caused by DNA/RNA bulges which may have structural implications. Inclusion of these additional features allows the models to better predict off-target activity and they outperform the CFD and MIT-Broad methods on independent datasets (Abadi et al., 2017; Listgarten et al., 2018). Supplementary Table 2 catalogs some of the more common off-target detection tools and summarizes their key differences.

## Future Perspective

A key goal of future research will be to improve the accuracy of predictive models by incorporating additional features. Current methods for predicting target efficiency and specificity are based solely on the sequence of the target site. However, it is now accepted that chromatin environment (Chari et al., 2015; Knight et al., 2015; Horlbeck et al., 2016b; Isaac et al., 2016; Chen et al., 2017) can influence CRISPR-Cas9 activity. Early studies mapping the genome wide binding of inert Cas9 enzymes using ChIP-seq showed a preference for DNAse sensitive regions (Kuscu et al., 2014; Wu et al., 2014; O’Geen et al., 2015), which are typically more accessible environments. This was supported by later studies which showed that high-activity target sites were often enriched for histone modifications associated with open-chromatin environments (Chari et al., 2015).

A direct link between chromatin and CRISPR-Cas9 activity was shown in 2016, where a pair of studies demonstrated that the presence of nucleosomes at the target site physically blocked CRISRP-Cas9’s access and reduced overall activity (Horlbeck et al., 2016b; Isaac et al., 2016). The differences in chromatin environment likely explain why the same CRISPR-Cas9 target site can display different activities across cell-lines (Chari et al., 2015). There is also evidence that off-target activity is influenced by chromatin accessibility, with the CROP-IT pipeline including this information into their off-target model (Singh et al., 2015). Incorporating environmental information in future predictive models will help improve accuracy and will be critical if the technology is to be applied in the clinic. Such modeling may also allow for the selective targeting of individual tissues by leveraging the differences in chromatin environments.

Incorporation of chromatin environments would likely also improve off-target predictions, which is thought to be more susceptible to chromatin accessibility. Besides chromatin information, future off-target pipelines should also focus on including variant information. A recent study demonstrated that the variance between individuals has a dramatic effect on the off-target landscape, with point mutations creating and destroying potential off-target sites (Lessard et al., 2017). Such information is critical for the application of CRISPR technology in almost all fields, as not taking an individual’s unique genome into account could have deleterious side-effects (Canver et al., 2017, 2018; Scott and Zhang, 2017).

Future models may also not only be able to predict the success of CRISPR-Cas9 editing, but also the outcome. By targeting sites with microhomology and exploiting the microhomology-mediated repair pathway, researchers may be able to delete specific DNA segments and thereby control the outcome of CRISPR-Cas9 editing (Bae et al., 2014a; Yao et al., 2017). Additionally, a recent study found that the mutations induced by repair of CRISPR-Cas9 cleavage were non-random and determined by the target sequence (van Overbeek et al., 2016). Such a finding suggests that it would be possible to predict the mutational outcome of CRISPR-Cas9 editing, allowing for researchers to make precise edits without the need of using knock-ins.

**FIGURE 1:**
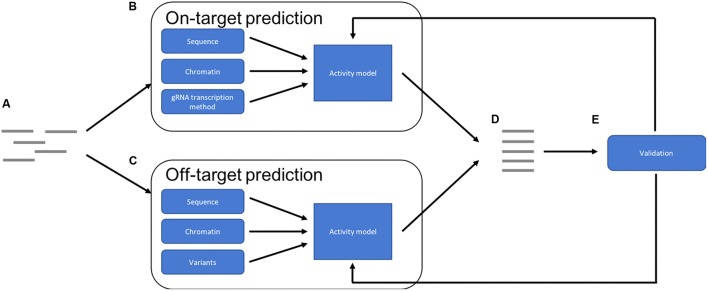
Overview of an optimal prediction pipeline: **(A)** potential target sites are identified. **(B)** On-target activity is predicted using a combination of target sequence, chromatin features and gRNA transcription method. **(C)** Off-target activity is predicted using a combination of sequence and chromatin features while also taking sequence variations into account. **(D)** The results are combined and then a ranked list of optimal targets can be provided. **(E)** The predictions can be validated experimentally and then used to improve accuracy of the models, improving future predictions.

The optimal future pipeline will incorporate all of these factors into both on- and off-target activity predictions (**Figure 1**). Such a pipeline could also provide a method by which experimentally validated predictions could be reintegrated into the training data for the models, to continue to improve accuracy. Future models may also predict success of other CRISPR-Cas9 applications such as knock-ins (Merkle et al., 2015), which involve the repair of the double strand break using a supplied template, and base-editing, where a Cas9 fusion protein converts one base into another without cleavage (Gaudelli et al., 2017).

## Conclusion

Computational tools for the prediction of CRISPR-Cas9 activity are necessary for the efficient design of experiments. However, current tools are hampered by a range of issues, such as disparate training data sources, which results in models not generalizing, as well as limitations in our current understand of factors that drive CRISPR-Cas9 activity. As our understanding improves, we will be able to incorporate new features into predictive models to increase their accuracy. This will be vital for applying CRISPR-Cas9 in clinical applications, where an individual’s genomic variations may alter activity patters of CRISPR-Cas9. Until then, it is important that the data used to train a predictive model is understood before it is used, to ensure models are only applied in appropriate circumstances.

## Author Contributions

LW researched the software highlighted in the paper. LW and DB designed and wrote the review paper. AO contributed to Section “Future Perspective.”

## Conflict of Interest Statement

The authors declare that the research was conducted in the absence of any commercial or financial relationships that could be construed as a potential conflict of interest.
