# Supplementary material for: The Current State and Future of CRISPR-Cas9 gRNA Design Tools
Source: Front Pharmacol. 2018 Jul 12;9:749. doi: 10.3389/fphar.2018.00749 (PMC6052051; doi:10.3389/fphar.2018.00749)
Supplement: Supplementary file 1 [file Table_1.DOCX]

| **Program name** | **Identifies/Scores targets?** | **Training Data used for model** | **gRNA transcription method** | **Model Features and Implementation** | **Comments** |
| --- | --- | --- | --- | --- | --- |
| SSFinder (Chari et al., 2015) | Identifies targets | NA | NA | NA | Does not score activity. Filters targets if the seed region (12bp preceding the PAM) are not unique within the input sequence. |
| sgRNAcas9 (Xie et al., 2014) | Identifies targets | NA | NA | NA | Does not score activity. Ranks targets based on potential off-targets (detected via alignment with SeqMap) |
| CRISPRseek (Zhu et al., 2014) | Identifies targets | NA | NA | NA | Does not score activity. Implemented as a Bioconductor package for R. |
| sgRNA Scorer (Chari et al., 2015) | Identifies and scores targets | Mutation rates at target sites in HEK293t cells following treatment with CRISPR-Cas9 and gRNAs | *In vitro* transcription using a U6 promoter | SVM model using nucleotide composition of target site of the target site | Standalone program. |
| Rule Set 1 (Doench et al., 2014) | Scores targets | Enrichment rates of transfected gRNA following selection for changes in expression of cell-surface markers as determined by FACS in human and mouse cells. | *In vitro* transcription using a U6 promoter | Logistic Regression model using nucleotide composition of the target site | No implementation is available however the feature weights are provided in the paper allowing for standalone implementation. |
| CRISPRscan (Moreno-Mateos et al., 2015) | Identifies and scores targets | Mutation rates at target sites in zebrafish embryos. | *In vitro* transcription using a T7 promoter | Linear Regression model using nucleotide composition of the target site | Implemented as a web-app (www.crisprscan.org). Features weights are also provided in the paper allowing for standalone implementations. |
| WU-CRISPR (Wong et al., 2015) | Identifies and scores targets | Reanalysis of the data from Doench 2014 | *In vitro* transcription using a U6 promoter | SVM model using nucleotide composition of the target site and sgRNA secondary structure | Employs strict filtering criteria which results in a majority of potential targets being discarded prior to scoring. Implemented as a standalone program. |
| Azimuth (Doench et al., 2016) | Identifies and scores targets | Enrichment rates of transfected sgRNAs targeting drug-resistance pathways following drug challenge in human cells. Also incorporates data from Doench 2014 | *In vitro* transcription using a U6 promoter | Combined SVM and Logistic Regression model using nucleotide composition of the target site and flanking region, sgRNA secondary structure and position of target site relative to transcription start | Implemented as a stand-alone program and as a Web-app (https://www.microsoft.com/en-us/research/project/crispr/) |
| TUSCAN (Wilson et al., 2018) | Identifies and scores targets | Reanalysis of the data from Chari 2015 | *In vitro* transcription using a U6 promoter | Random Forest model using nucleotide composition of the target site and flanking region | Provides both activity score and general active/inactive classification. Implemented as a stand-alone program and as a Web-app (https://www.gt-scan.net/tuscan) |

**Table 1: Summary of selected CRISPR-Cas9 activity models**

References

Chari, R., Mali, P., Moosburner, M., and Church, G. M. (2015). Unraveling CRISPR-Cas9 genome engineering parameters via a library-on-library approach. *Nat. Methods* 12, 823–826. doi:10.1038/nmeth.3473.

Doench, J. G., Fusi, N., Sullender, M., Hegde, M., Vaimberg, E. W., Donovan, K. F., Smith, I., Tothova, Z., Wilen, C., Orchard, R., et al. (2016). Optimized sgRNA design to maximize activity and minimize off-target effects of CRISPR-Cas9. *Nat. Biotechnol.* 34, 184–191. doi:10.1038/nbt.3437.

Doench, J. G., Hartenian, E., Graham, D. B., Tothova, Z., Hegde, M., Smith, I., Sullender, M., Ebert, B. L., Xavier, R. J., and Root, D. E. (2014). Rational design of highly active sgRNAs for CRISPR-Cas9-mediated gene inactivation. *Nat. Biotechnol.* 32, 1262–1267. doi:10.1038/nbt.3026.

Moreno-Mateos, M. A., Vejnar, C. E., Beaudoin, J.-D., Fernandez, J. P., Mis, E. K., Khokha, M. K., and Giraldez, A. J. (2015). CRISPRscan: designing highly efficient sgRNAs for CRISPR-Cas9 targeting in vivo. *Nat. Methods* 12, 982–988. doi:10.1038/nmeth.3543.

Wilson, L. O. W., Reti, D., O’Brien, A. R., Dunne, R. A., and Bauer, D. C. (2018). High Activity Target-Site Identification Using Phenotypic Independent CRISPR-Cas9 Core Functionality. *The CRISPR Journal* 1, 182–190. doi:10.1089/crispr.2017.0021.

Wong, N., Liu, W., and Wang, X. (2015). WU-CRISPR: characteristics of functional guide RNAs for the CRISPR/Cas9 system. *Genome Biol.* 16, 218. doi:10.1186/s13059-015-0784-0.

Xie, S., Shen, B., Zhang, C., Huang, X., and Zhang, Y. (2014). sgRNAcas9: a software package for designing CRISPR sgRNA and evaluating potential off-target cleavage sites. *PLoS One* 9, e100448. doi:10.1371/journal.pone.0100448.

Zhu, L. J., Holmes, B. R., Aronin, N., and Brodsky, M. H. (2014). CRISPRseek: a bioconductor package to identify target-specific guide RNAs for CRISPR-Cas9 genome-editing systems. *PLoS One* 9, e108424. doi:10.1371/journal.pone.0108424.
