# Supplementary material for: The Current State and Future of CRISPR-Cas9 gRNA Design Tools
Source: Front Pharmacol. 2018 Jul 12;9:749. doi: 10.3389/fphar.2018.00749 (PMC6052051; doi:10.3389/fphar.2018.00749)
Supplement: Supplementary file 2 [file Table_2.DOCX]

| Program name | Off-target detection method | Off-target scoring method | Notes |
| --- | --- | --- | --- |
| GT-Scan (O’Brien and Bailey 2014) | Alignment with Bowtie2 | NA | Allows user to identify the required sequence rules (e.g., what PAMs are acceptable, how large a target site). Available as a Web-app (https://www.gt-scan.net/) |
| CCTop (Stemmer et al. 2015) | Alignment with Bowtie | Custom model based on presence of mismatches and weather they fall within seed region. | Allows for selection of various canonical PAMs and some sequence limitations. Available as a Web-app (https://crispr.cos.uni-heidelberg.de/) |
| CROP-IT (Singh et al. 2015) | Alignment with PATMAN | Custom model based on position of mismatches and also weather the potential off-target falls within a DNase-sensitive region | Allows for selection between NGG or NNG PAMs. Available as a Web-app (http://cheetah.bioch.virginia.edu/AdliLab/CROP-IT/homepage.html) |
| CRISPOR (Haeussler et al. 2016) | Alignment with BWA | MIT-Broad score | Allows for selection of various canonical PAMs. Available as a Web-app (http://crispor.tefor.net/) |
| Elevation (Listgarten et al. 2018) | Custom alignment tool | Custom model based on the number, position and type (wobble vs bulge) of mismatches. | Available as a web-app (crispr.ml). The scoring method is available as a stand-alone tool |
| CRISTA (Abadi et al. 2017) | NA | Custom model based on the number, position and type (wobble vs bulge) of mismatches. | Available as a stand-alone tool |

**Table 2: Summary of selected off-target predictor tools**

Bibliography

Abadi, S., Yan, W.X., Amar, D. and Mayrose, I. 2017. A machine learning approach for predicting CRISPR-Cas9 cleavage efficiencies and patterns underlying its mechanism of action. *PLoS Computational Biology* 13(10), p. e1005807.

Haeussler, M., Schönig, K., Eckert, H., et al. 2016. Evaluation of off-target and on-target scoring algorithms and integration into the guide RNA selection tool CRISPOR. *Genome Biology* 17(1), p. 148.

Listgarten, J., Weinstein, M., Kleinstiver, B.P., et al. 2018. Prediction of off-target activities for the end-to-end design of CRISPR guide RNAs. *Nature biomedical engineering* 2(1), pp. 38–47.

O’Brien, A. and Bailey, T.L. 2014. GT-Scan: identifying unique genomic targets. *Bioinformatics* 30(18), pp. 2673–2675.

Singh, R., Kuscu, C., Quinlan, A., Qi, Y. and Adli, M. 2015. Cas9-chromatin binding information enables more accurate CRISPR off-target prediction. *Nucleic Acids Research* 43(18), p. e118.

Stemmer, M., Thumberger, T., Del Sol Keyer, M., Wittbrodt, J. and Mateo, J.L. 2015. Cctop: an intuitive, flexible and reliable crispr/cas9 target prediction tool. *Plos One* 10(4), p. e0124633.
